# Impact of risk factor burden and vascular bed involvement on dementia risk in individuals with established cardiovascular disease

**DOI:** 10.1016/j.ajpc.2026.101639

**Published:** 2026-04-17

**Authors:** Jan F. de Leijer, Geert Jan Biessels, Frank L.J. Visseren, Tessa A.C.M. Vissers, Robin W.M. Vernooij, Frank J. Wolters, Sanaz Sedaghat, Pamela L. Lutsey, Mike J.L. Peters, Archana Singh-Manoux, Thomas T. van Sloten, UCC-SMART study group

**Affiliations:** a Department of Vascular Medicine & Endocrinology, University Medical Center Utrecht, Utrecht, the Netherlands; b Department of Neurology, UMC Utrecht Brain Center, University Medical Center Utrecht, Utrecht, the Netherlands; c Department of Nephrology and Hypertension, University Medical Center Utrecht, Utrecht, the Netherlands; d Department of Epidemiology, Erasmus MC University Medical Center, Rotterdam, the Netherlands; e Department of Radiology and Nuclear Medicine and Alzheimer Center, Erasmus MC University Medical Centre, Rotterdam, the Netherlands; f Division of Epidemiology and Community Health, School of Public Health, University of Minnesota, Minneapolis, USA; g Department of Geriatrics, University Medical Center Utrecht, Utrecht, the Netherlands; h Université Paris Cité, Inserm U1153, Epidemiology of Ageing and Neurodegenerative Diseases, Paris, France; i Faculty of Brain Sciences, University College London, London, UK

**Keywords:** Dementia, Cardiovascular disease, Risk factor burden, Vascular bed involvement, Cohort study

## Abstract

**Aim:**

To determine how cardiovascular disease (CVD) risk factor burden and vascular bed involvement relate to the risk of dementia in individuals with established CVD.

**Methods:**

Participants were from the prospective, single-center UCC-SMART cohort study, recruited between 1996–2024. CVD risk factor burden was defined as the number of CVD risk factors present. The number of affected vascular beds was the count of coronary artery disease, cerebrovascular disease, and peripheral artery disease. Relations with all-cause dementia were estimated with Cox regression adjusted for confounders.

**Results:**

Among 10,321 participants (mean age 61±10 years; 27% female), 296 developed dementia during a median follow-up of 10.1 years (IQR 5.0–15.7). Dementia risk increased with higher CVD risk factor burden: compared with 0–1 risk factors, hazard ratios were 1.35 (95% CI 0.95–1.92) for 2 risk factors, 1.58 (95% CI 1.10–2.26) for 3 risk factors, and 1.87 (95% CI 1.31–2.67) for 4–9 risk factors. Compared with 1 affected vascular bed, the hazard ratios were 1.32 (95% CI 1.02–1.71) for 2 affected vascular beds and 1.90 (95% CI 1.05–3.43) for 3 affected vascular beds. Dementia risk was higher in participants with peripheral artery disease (HR 1.68; 95% CI 1.24–2.28) and cerebrovascular disease (HR 2.09; 95% CI 1.61–2.71) than in those with coronary artery disease.

**Conclusion:**

Higher CVD risk factor burden and involvement of multiple vascular beds are related to increased dementia risk in individuals with established CVD. These findings underscore the importance of stringent cardiovascular risk management as a potential strategy to lower dementia risk in this population.

## 1 Introduction

Individuals with established cardiovascular disease (CVD) have up to twice the risk of late-onset dementia compared to those without established CVD[1]. This higher risk is present among all major clinical manifestations of CVD and is most pronounced for cerebrovascular disease [[2], [3], [4]]. Prevalence of CVD is expected to double to over 1.1 billion affected individuals in 2050, mainly driven by population growth and ageing [5]. At the same time, loss of life expectancy due to CVD has decreased by 35%−55% over the past decades and is expected to decrease further in the next decades [[5], [6], [7]]. As the number of older individuals living with CVD continues to rise, the long-term complications of CVD, notably dementia, are becoming increasingly important. In the absence of curative therapies for dementia, prevention of dementia in individuals with established CVD is therefore of vital importance.

Population-based studies have consistently shown a higher CVD risk factor burden to be related to higher risk of dementia among individuals without established CVD [8,9]. Accordingly, the European Society of Cardiology[10] and the American Heart Association[11] have highlighted cardiovascular risk management as an important strategy for the prevention of late-onset dementia. It remains unclear whether CVD risk factor burden is also related to dementia risk in individuals with established CVD. In these individuals, the impact of CVD risk factors on dementia risk may also depend on ongoing vascular damage and iatrogenic effects. Furthermore, the involvement of different and multiple vascular beds may play a role in shaping risk of late-onset dementia. Understanding the relations of CVD risk factor burden and affected vascular beds with dementia risk could inform personalized management strategies, allowing for more targeted monitoring and intervention in high-risk individuals.

Accordingly, the aim of this study was to quantify the relation of CVD risk factor burden with dementia risk in individuals with established CVD. We also examined whether dementia risk varied according to vascular bed involvement, both for the number of affected vascular beds and for individual vascular beds.

## 2 Methods

### 2.1 Study population

Participants in this study were drawn from the Utrecht Cardiovascular Cohort-Second Manifestations of ARTerial disease (UCC-SMART) study, an ongoing prospective cohort study of individuals with established CVD or CVD risk factors at the University Medical Center Utrecht, the Netherlands. The rationale and design have been described in detail elsewhere [12]. In brief, individuals aged 18–90 referred to University Medical Center Utrecht for management of CVD or CVD risk factors were recruited after their first visit to the outpatient clinic or inpatient unit. Individuals with a short life expectancy or prevalent dementia, as judged by their treating physician, were not recruited. At baseline, participants underwent a standardized vascular screening protocol. This was done at least 2 months after the qualifying cardiovascular event or intervention, when participants had reached a stable clinical condition. The study was approved by the local medical ethics committee (reference number 22–088) and written informed consent was obtained from all participants.

For the current analysis, all participants enrolled in UCC-SMART between September 1996 and January 2024 with established CVD at baseline were included. Established CVD was defined as coronary artery disease (i.e., myocardial infarction, angina pectoris, ≥1 vessel disease on coronary angiography, or coronary revascularization in medical history), cerebrovascular disease (i.e., transient ischemic attack, cerebral infarction, carotid artery stenosis, ischemic retinal syndrome, carotid surgery or carotid angioplasty in medical history), or peripheral artery disease (i.e., Fontaine classification ≥II with ankle-brachial index ≤0.9, amputation, vascular surgery or angioplasty in medical history). Detailed definitions of established CVD have been described previously [12].

### 2.2 CVD risk factor burden

CVD risk factor burden was defined for each patient as an unweighted sum of nine CVD risk factors, with each risk factor contributing one point if present. These risk factors were selected a priori, based on prior studies[[13], [14], [15], [16], [17]] that have shown them to be related to a higher risk of dementia. These risk factors were type 2 diabetes, current smoking, excessive alcohol use, elevated systolic blood pressure, elevated low-density lipoprotein (LDL) cholesterol, obesity, physical inactivity, elevated C-reactive protein (CRP), and albuminuria. Unhealthy diet was not included, as dietary data were only available for a subsample (26%) of the study population due to these being collected only after 2022.

Type 2 diabetes was defined as a fasting glucose plasma concentration of ≥7.0 mmol/L or non-fasting glucose plasma concentration of ≥11.1 mmol/L, a self-reported history or referral diagnosis of type 2 diabetes, or the use of glucose-lowering medication. Smoking status, alcohol use and physical activity in metabolic equivalent of task hours per week (METh/week) were obtained with health questionnaires. Excessive alcohol use was defined as ≥10 units per week. Blood pressure was measured bilaterally three times and mean systolic and diastolic blood pressure were calculated for the arm yielding the highest values. Waist circumference was measured midway between the iliac crest and lower costal margin, and hip circumference at the widest point over the gluteal region, with the mean of two measurements used. Waist-to-hip ratio was calculated by dividing waist by hip circumference. We used waist-to-hip ratio instead of BMI, as it is thought to be a better measure of obesity and less affected by weight loss in the preclinical phase of dementia [18,19]. A venous blood sample was drawn after at least 8 h of fasting to measure CRP and LDL-cholesterol. From 1996 until 2013 high-sensitivity CRP was measured by immunonephelometry and from 2013 on it was determined in heparin plasma using a routine chemistry analyzer. A morning-void urine sample was collected to determine urine albumin/creatinine ratio (UACR).

### 2.3 Outcome

The outcome of this study was incident all-cause dementia. Dementia status was ascertained with a combination of self-report questionnaires and review of hospital and primary care records. In the UCC-SMART cohort, participants are asked annually by mail to complete a questionnaire on any hospitalizations and outpatient clinic visits. From 2021 onward, all participants were asked whether they had been diagnosed with dementia. If participants or their proxies responded positively, further details regarding the date of diagnosis, dementia subtype, and diagnosing specialist were requested through an additional questionnaire. Dementia was only recorded if the diagnosis was verified in this additional questionnaire or in hospital medical records by the study team. Additionally, for all deceased participants (30% of study population), dementia status was verified retrospectively through review of medical records of available hospital and primary care records at the time of death. Dementia was only recorded if diagnosed by a neurologist, a general practitioner, or a geriatrician. All medical records were adjudicated by two medical doctors (J.F.d.L. or T.A.C.M.V.), with any uncertainties resolved by an independent internal medicine specialist (T.T.v.S.). In case of a possible interim stroke (ischemic or hemorrhagic), other cardiovascular event, or death, all available data were retrieved and adjudicated by the UCC-SMART endpoint committee, consisting of three independent physicians. Participants were followed for all outcomes from time of cohort entry until death, dementia or the predetermined end-of-study date of January 1, 2024 (i.e., the most recent date with complete collection and adjudication of outcomes). Follow-up was complete in 92.1% of participants.

### 2.4 Covariates

Education level was classified into three categories: low (lower secondary education or below), middle (upper secondary or post-secondary non-tertiary education), and high (tertiary education or above). Medication use was self-reported at baseline. Atrial fibrillation was assessed at baseline using a protocolised 12-lead resting electrocardiogram. APOE genotype (rs7412 and rs429358) was also assessed.

### 2.5 Statistical analyses

Missing data were imputed using multiple imputation by chained equations (50 imputations, 20 iterations) and convergence was assessed visually using trace plots. APOE genotype was measured only in participants enrolled between cohort inception and 2010, and was consequently missing in the 4777 participants (46%) enrolled after 2010. Education level data were collected from 2004 onwards only and were missing in 3251 participants (32%). Waist-to-hip ratio was missing in 1033 participants (10%), and all other variables had ≤4.0% missing data.

Baseline characteristics were described according to CVD risk factor burden and the number of affected vascular beds (i.e., coronary artery disease, cerebrovascular disease, and peripheral artery disease, as described in the Study population section). To examine the relation between CVD risk factor burden and incident dementia, participants were divided into four groups of approximately equal size (i.e., 0–1 risk factors, 2 risk factors, 3 risk factors, and 4–9 risk factors), with the group having 0–1 CVD risk factors serving as the reference. Risk factors measured on a continuous scale contributed to the CVD risk factor burden if values were in the highest quartile of systolic blood pressure (≥150 mmHg), LDL-cholesterol (≥3.3 mmol/L), waist-to-hip ratio (sex-specific: ≥0.9 for women, ≥1.0 for men), CRP (≥4.3 mg/L), and UACR (≥1.7 mg/mmol), or in the lowest quartile of physical activity (≤16 METh/week). Systolic blood pressure and LDL-cholesterol were dichotomized irrespective of baseline medication use. The relation of individual vascular beds and the number of affected vascular beds with incident dementia was examined using participants with coronary artery disease and those with one affected vascular bed as reference, respectively. Cause-specific Cox proportional hazards models with age as the time scale were used to estimate relations of CVD risk factor burden, the number of affected vascular beds, and their individual components, with incident dementia. These models account for the competing risk of mortality by censoring those who died over the follow-up at time of death. The number of affected vascular beds was modelled as a time-varying covariate whenever an additional vascular bed became affected after baseline. Accordingly, each participant’s follow-up time was split into intervals defined by exposure status, and estimates were derived as a weighted average across all intervals. Analyses were first adjusted for age (as the time scale, Model 1), and further adjusted for sex, education level, APOE genotype, years since first CVD diagnosis, atrial fibrillation, and use of blood pressure-lowering, lipid-lowering, and antithrombotic medication (Model 2). Analyses with individual CVD risk factors as the determinant were additionally mutually adjusted (Model 3). We obtained estimates using Rubin’s rules to pool the results from each imputed dataset. The proportional hazards assumption was assessed visually using Schoenfeld residuals and was not violated. Linearity of relations between continuous determinants and incident dementia was evaluated both visually and formally using restricted cubic splines and was not violated. Cumulative incidence function curves adjusted for age and sex were calculated stratified by CVD risk factor burden and number of affected vascular beds using cause-specific Cox models.

We undertook several additional analyses. The relations of the number of CVD risk factors and continuous individual CVD risk factors (i.e., systolic blood pressure, LDL-cholesterol, waist-to-hip ratio, physical activity, CRP, and UACR) with incident dementia were also examined using restricted cubic splines with three knots placed at the 10th, 50th, and 90th percentiles of the exposure distribution for insight into the shape of the relation. Three knots were selected based on the Akaike information criterion (AIC) [20]. Continuous measures of CRP and UACR were log-transformed in this analysis to account for their right-skewed distributions. Next, we quantified the relation between CVD risk factor burden and dementia using standard clinical cut-offs for individual risk factors instead of quartiles (i.e., ≥140 mmHg for systolic blood pressure, ≥1.8 mmol/L for LDL-cholesterol, ≥0.90 and ≥0.85 for waist-to-hip ratio in men and women, respectively, ≤10 METh/week for physical activity, ≥3 mg/L for CRP, and ≥3 mg/mmol for UACR) [10,21]. Additionally, we explored if the relation between CVD risk factor burden and incident dementia was mediated through the occurrence of an interim stroke, by adjusting Model 2 for interim stroke as a time-varying covariate.

Several sensitivity analyses were undertaken. To assess the potential impact of missing data on APOE genotype, we repeated the analyses in participants with complete data on APOE genotype with and without adjustment for APOE genotype. To explore effect modification by age at CVD onset, sex, vascular bed affected at baseline and APOE genotype, multiplicative interaction terms were added in separate models and likelihood ratio tests were used to examine whether inclusion of the interaction term significantly improved model fit. To evaluate the possibility of reverse causality, we repeated the analyses after excluding participants who were diagnosed with dementia within the first 1, 5 and 10 years after baseline, respectively. To investigate the impact of baseline medication use, we repeated the analyses after adjusting systolic blood pressure and LDL-cholesterol for expected effects of blood pressure-lowering and lipid-lowering therapies [22,23]. Systolic blood pressure was reduced by 5.7–31.7 mmHg depending on the amount of blood pressure-lowering medications used and unadjusted systolic blood pressure [23]. LDL-cholesterol was adjusted assuming a 24–49% reduction depending on type and dose of lipid-lowering therapy [22]. To examine the contribution of prior stroke, we repeated the analyses after excluding participants with a history of stroke at baseline. To assess potential temporal cohort effects, we repeated the analyses with additional adjustment for year of inclusion as a covariate. In addition, to account for the competing risk of all-cause mortality, we repeated the analyses using Fine and Gray subdistribution hazard models.

All analyses were conducted using R version 4.3.2 (R Foundation for Statistical Computing, Vienna, Austria). A p-value <0.05 was considered statistically significant.

## 3 Results

### 3.1 Study population

A total of 10,321 participants with CVD were included in the present study, of whom 6480 (63%) had coronary artery disease, 3378 (33%) cerebrovascular disease, and 2681 (26%) peripheral artery disease. Mean age of participants was 61 ± 10 years, and 7531 (73%) were male (Table 1). A flowchart of study enrollment is shown in **Supplemental Figure 1**. Prevalence of subcategories of baseline CVD is shown in **Supplemental Table 1**. A total of 2669 (26%) of participants had ≤1 CVD risk factor and 2537 (25%) had 4 to 9 risk factors. Those with higher CVD risk factor burden more often had a low education level and used lipid-lowering and antithrombotic medication less often. Prevalence of dichotomized individual CVD risk factors according to CVD risk factor burden categories is shown in **Supplemental Figure 2**. All CVD risk factors became more common with increasing CVD risk factor burden. Baseline characteristics of participants according to number of affected vascular beds are shown in **Supplemental Table 2**. Participants with multiple affected vascular beds more often had a higher CVD risk factor burden (**Supplemental Figure 3**). For example, 70% of participants with three affected vascular beds had three or more CVD risk factors.

**Table 1:** Baseline characteristics according to CVD risk factor burden.

	Overall (n = 10,321)	0–1 risk factors (n = 2669)	2 risk factors (n = 2764)	3 risk factors (n = 2351)	4–9 risk factors (n = 2537)
Male sex	7531 (73)	2015 (76)	2045 (74)	1690 (72)	1781 (70)
Age (years)	61 ± 10	60 ± 11	61 ± 10	61 ± 10	62 ± 10
Current alcohol use	6028 (58)	1323 (50)	1764 (64)	1454 (62)	1487 (59)
Current smoking	2900 (28)	147 (6)	604 (22)	847 (36)	1302 (51)
Packyears	13 (1 - 31)	5 (0 - 19)	11 (0 - 27)	18 (5 - 33)	24 (10 - 39)
Physical activity (METh/week)	45 ± 41	55 ± 40	50 ± 41	43 ± 41	30 ± 36
Education level					
Low	3425 (33)	707 (27)	865 (31)	835 (36)	1018 (40)
Middle	3515 (34)	877 (33)	910 (33)	826 (35)	902 (36)
High	3381 (33)	1085 (41)	989 (36)	690 (29)	617 (24)
Affected vascular beds					
Coronary artery disease	6480 (63)	1947 (73)	1857 (67)	1373 (58)	1303 (51)
Cerebrovascular disease	3378 (33)	752 (28)	865 (31)	842 (36)	919 (36)
Peripheral artery disease	2681 (26)	309 (12)	504 (18)	696 (30)	1172 (46)
≥2 affected vascular beds	1953 (19)	307 (12)	414 (15)	499 (21)	733 (29)
Type 2 diabetes	1813 (18)	86 (3)	303 (11)	454 (19)	970 (38)
Medication use					
Blood pressure-lowering	7922 (77)	2089 (78)	2135 (77)	1766 (75)	1932 (76)
Lipid-lowering	7438 (72)	2202 (83)	2108 (76)	1595 (68)	1533 (60)
Antithrombotic	8700 (84)	2404 (90)	2398 (87)	1920 (82)	1978 (78)
Anthropometric measurements					
Body mass index (kg/m^2^)	27 ± 4	26 ± 4	27 ± 4	27 ± 4	28 ± 5
Waist-to-hip ratio (F)	0.85 ± 0.08	0.82 ± 0.06	0.84 ± 0.07	0.86 ± 0.07	0.89 ± 0.08
Waist-to-hip ratio (M)	0.95 ± 0.07	0.91 ± 0.05	0.94 ± 0.06	0.96 ± 0.07	0.99 ± 0.07
Systolic blood pressure (mmHg)	138 ± 21	129 ± 14	135 ± 18	141 ± 21	150 ± 23
Diastolic blood pressure (mmHg)	81 ± 11	78 ± 10	80 ± 11	82 ± 11	84 ± 13
APOE genotype					
One ε4 allele	3540 (34)	1016 (38)	943 (34)	787 (34)	794 (31)
Two ε4 alleles	219 (2)	54 (2)	64 (2)	49 (2)	52 (2)
Laboratory measurements					
eGFR (ml/min/1.73m^2^)	82 ± 18	85 ± 16	83 ± 17	82 ± 19	78 ± 22
UACR (mg/mmol)	0.8 (0.5 - 1.7)	0.6 (0.4 - 0.9)	0.7 (0.4 - 1.3)	0.9 (0.5 - 2.0)	2.0 (0.8 - 5.0)
HbA1c (%)	5.7 (5.4 - 6.0)	5.5 (5.3 - 5.8)	5.6 (5.4 - 5.9)	5.7 (5.4 - 6.0)	5.9 (5.6 - 6.6)
Total cholesterol (mmol/L)	4.7 ± 1.2	4.2 ± 0.9	4.5 ± 1.1	4.8 ± 1.2	5.2 ± 1.4
HDL-cholesterol (mmol/L)	1.2 ± 0.4	1.3 ± 0.3	1.3 ± 0.4	1.2 ± 0.4	1.2 ± 0.4
LDL-cholesterol (mmol/L)	2.7 ± 1.1	2.3 ± 0.7	2.6 ± 1.0	2.8 ± 1.1	3.1 ± 1.2
C-reactive protein (mg/L)	2.0 [1.0 - 4.3]	1.2 [0.7 - 2.1]	1.6 [0.9 - 3.1]	2.5 [1.2 - 5.0]	4.7 [2.0 - 8.0]

Values are median (IQR), mean ± SD or count (%).

Abbreviations: CVD - cardiovascular disease, eGFR - estimated glomerular filtration rate, HbA1c - glycated haemoglobin, HDL - high-density lipoprotein, LDL - low-density lipoprotein, M - male, METh/week - metabolic equivalent of task hours per week, F - female, UACR - urine albumin/creatinine ratio.

### 3.2 Relation between CVD risk factor burden and dementia risk

**Fig. 1:**
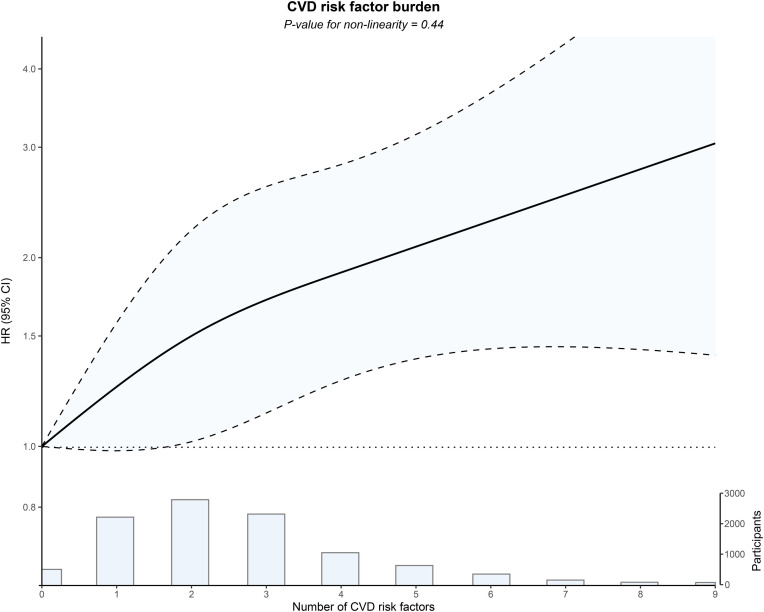
Dementia risk as a function of continuously increasing number of CVD risk factors, modeled using restricted cubic splines (3 knots), with 0 risk factors as reference (HR: 1.0). The histogram shows the corresponding distribution of participants. The spline is adjusted for age, sex, education level, APOE genotype, years since first CVD diagnosis, atrial fibrillation, and blood pressure-lowering, lipid-lowering and antithrombotic medication (Model 2).

**Fig. 2:**
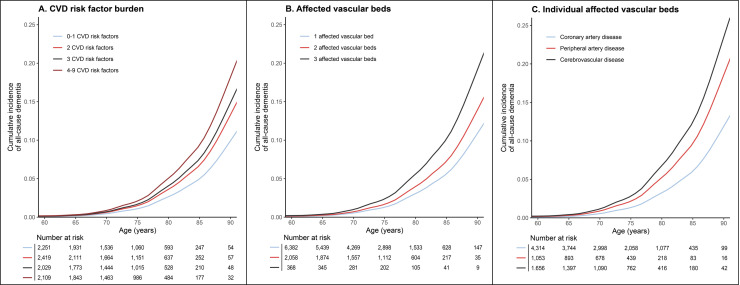
Shown is the age- and sex-adjusted cumulative incidence of all-cause dementia among individuals with established CVD according to (A) CVD risk factor burden, (B) number of affected vascular beds and (C) individual affected vascular beds. Curves were derived from cause-specific Cox models.

A total of 296 incident dementia cases were observed during a median follow-up of 10.1 years (IQR: 5.0–15.7). The incidence rate of dementia was 2.7 per 1000 person-years. A higher CVD risk factor burden was related to a higher risk of dementia (Fig. 1), with no evidence of a non-linear relation between the number of CVD risk factors and dementia risk (p-value for non-linearity = 0.44). Age- and sex-adjusted cumulative incidence rose with higher CVD risk factor burden (Fig. 2). Compared to participants with 0–1 CVD risk factors, the risk of dementia was not statistically significantly higher in those with 2 risk factors (HR 1.35; 95% CI: 0.95–1.92), but was higher in those with 3 risk factors (HR 1.58; 95% CI: 1.10–2.26), and those with 4–9 risk factors (HR 1.87; 95% CI: 1.31–2.67) (Table 2). Of the individual risk factors, only the relation of type 2 diabetes with higher dementia risk (HR 1.46; 95% CI: 1.10–1.92) was statistically significant (**Supplemental Table 3**). The hazard ratios were similar when using standard clinical cut-offs for individual risk factors instead of quartile cut-offs (**Supplemental Table 4**). Restricted cubic splines of the relations of CVD risk factors considered as continuous variables and dementia are shown in **Supplemental Figure 4**. No evidence of non-linearity was found (p-values for non-linearity ≥0.10).

**Table 2:** Relation between CVD risk factor burden, vascular bed involvement, and incident dementia.

	All-cause dementia (296 events / 10,321 individuals / 107,977 person-years)			
	Events / individuals	Events / 1000 person-years	Model 1 HR (95% CI)	Model 2 HR (95% CI)
**CVD risk factor burden**				
0–1 risk factors	52 / 2669	1.79	ref	ref
2 risk factors	77 / 2764	2.59	1.34 (0.94 - 1.90)	1.35 (0.95 - 1.92)
3 risk factors	78 / 2351	3.14	1.61 (1.12 - 2.30)	1.58 (1.10 - 2.26)
4–9 risk factors	89 / 2537	3.66	1.90 (1.35 - 2.69)	1.87 (1.31 - 2.67)
**Number of affected vascular beds**				
1 affected vascular bed	227 / 8368*	2.50*	ref	ref
2 affected vascular beds	57 / 1688*	3.76*	1.31 (1.02 - 1.69)	1.32 (1.02 - 1.71)
3 affected vascular beds	12 / 265*	6.01*	1.77 (0.99 - 3.19)	1.90 (1.05 - 3.43)
**Individual affected vascular beds**				
Coronary artery disease	98 / 4987	1.82	ref	ref
Peripheral artery disease	39 / 1280	2.72	1.65 (1.25 - 2.17)	1.68 (1.24 - 2.28)
Cerebrovascular disease	90 / 2101	3.96	2.08 (1.62 - 2.65)	2.09 (1.61 - 2.71)

Hazard ratios with 95% confidence intervals as derived from Cox proportional hazards models adjusted for age (Model 1) and with additional adjustment for sex, education level, APOE genotype, years since first CVD diagnosis, atrial fibrillation, and blood pressure-lowering, lipid-lowering and antithrombotic medication (Model 2). Analyses of individual vascular beds were restricted to participants with one affected vascular bed.

⁎ Values represent participants at baseline. Because the number of affected vascular beds was modeled as a time-varying covariate, participants could contribute follow-up time to multiple groups if additional vascular beds became affected during follow-up. Abbreviations: CVD - cardiovascular disease.

Interim stroke was observed in 592 patients (5.7%), of whom 38 (6.4%) developed dementia after a median of 3.2 years (IQR: 1.9–5.2) post-stroke. Interim stroke strongly increased the risk of subsequent incident dementia (HR 2.43; 95% CI: 1.64–3.60). However, the relation between CVD risk factor burden and incident dementia was not attenuated by additional adjustment for interim stroke (**Supplemental Table 5**).

### 3.3 Relation between the number of affected vascular beds and dementia risk

Compared to participants with 1 affected vascular bed, a higher risk of dementia was found in those with 2 affected vascular beds (HR 1.32; 95% CI: 1.02–1.71), and in those with 3 affected vascular beds (HR 1.90; 95% CI: 1.05–3.43) (Table 2). Analyses of the three vascular beds individually among individuals with one affected vascular bed showed a higher risk of dementia in participants with cerebrovascular disease (HR 2.09; 95% CI: 1.61–2.71) and peripheral artery disease (HR 1.68; 95% CI: 1.24–2.28) compared to participants with coronary artery disease (Table 2).

### 3.4 Sensitivity analyses

Results were consistent when analyses were restricted to participants with data on APOE genotype available, both before and after adjustment for APOE genotype (**Supplemental Table 6**). No evidence of effect modification by age at CVD onset, sex, baseline affected vascular bed or APOE genotype was found (p-values for interaction ≥0.11) (**Supplemental Table 7–10**). Hazard ratios were not substantially different when participants who were diagnosed with dementia within 1, 5, or 10 years after baseline were excluded from the analyses (**Supplemental Table 11**). Results were similar when we adjusted systolic blood pressure and LDL-cholesterol for medication use (**Supplemental Table 12**). Hazard ratios were slightly attenuated when repeating analyses after excluding participants with stroke as qualifying event for inclusion or a history of stroke at baseline (**Supplemental Table 13**). Results were unchanged after additional adjustment for year of inclusion as a covariate (**Supplemental Table 14**). Results were broadly similar in Fine and Gray models accounting for the competing risk of all-cause mortality (**Supplemental Table 15**).

## 4 Discussion

In this prospective cohort study of 10,321 participants with established CVD drawn from a setting with access to good healthcare for cardiovascular risk management, a higher CVD risk factor burden was related to a higher risk of dementia, with the clearest increase observed from 2 risk factors onward. Similarly, a higher number of affected vascular beds was related to a higher risk of dementia, driven by the increased risk of dementia in those with peripheral artery and cerebrovascular disease.

In line with these findings, three previous studies found CVD risk factor burden to be related to dementia risk in individuals with established CVD [[24], [25], [26]]. Two of these studies used a more specific definition of CVD, limited to coronary artery disease or ischemic stroke [24,26]. In addition, two of these studies focused on lifestyle risk factors (i.e., overweight, smoking, physical inactivity, and unhealthy diet) only [25,26]. The current findings extend this evidence by demonstrating that the relation between CVD risk factor burden and subsequent dementia persists even among individuals with a broader spectrum of established CVD and had a higher statistical power than previous studies (i.e., earlier studies had 59 and 115 incident dementia cases compared to 296 cases in the present study). Furthermore, we found that individuals with CVD who had an adverse profile on a more comprehensive set of risk factors, including lifestyle and non-lifestyle factors, had a higher risk of dementia.

Participants with multiple affected vascular beds were at the highest risk of dementia. This may in part be explained by shared cardiometabolic risk factors for CVD and dementia, but is likely also to be due to the cumulative impact of CVD on brain health. CVD is thought to contribute to dementia through multiple mechanisms, including cerebrovascular ischemia caused by atherosclerosis, emboli or small vessel disease, and cerebral hypoperfusion resulting from heart failure or carotid artery stenosis [27,28]. Other mechanisms related to CVD that may play a role include systemic inflammation, oxidative stress, and microvascular dysfunction [27,28]. In our study, participants with cerebrovascular disease and peripheral artery disease had a higher risk of dementia compared to participants with coronary artery disease. Brain damage resulting from cerebrovascular events is a well-established risk factor for cognitive impairment [[1], [2], [3], [4]]. In contrast, the link with peripheral artery disease may be more indirect, reflecting greater cumulative exposure to cardiovascular risk factors and a higher burden of systemic atherosclerosis than coronary artery disease [1,2].

Among individual CVD risk factors, type 2 diabetes was most strongly related to a higher risk of dementia. This suggests that prevention of type 2 diabetes may be particularly important for reducing dementia risk in individuals with CVD. It may, in part, also reflect the dynamics of risk factors across the lifespan and how we defined them [29]. Type 2 diabetes typically occurs in the context of other risk factors that are often present before the onset of diabetes, and when diabetes develops, hyperglycaemia may add to pre-existing vascular injury. Moreover, once diagnosed with type 2 diabetes, individuals were always considered as having type 2 diabetes in our study, regardless of their treated HbA1c levels. By contrast, those with adequately treated systolic blood pressure and LDL-cholesterol with prior hypertension or hypercholesterolemia may have been included in the low blood pressure or cholesterol group. This may explain the absence of a relation of systolic blood pressure and LDL-cholesterol with dementia risk in this study, as baseline measurements may not adequately reflect true exposure prior to baseline and during follow-up. Even though our results were similar after adjusting systolic blood pressure and LDL-cholesterol for medication use at baseline, treated and untreated values of systolic blood pressure and LDL-cholesterol remain difficult to compare. It may also be explained in part by the long prodromal phase of dementia, during which these measures tend to decline. This could lead to an apparent protective relation of these risk factors with dementia due to reverse causation [30,31]. Although our analysis with exclusion of dementia cases within the first 1, 5, or 10 years after baseline did not change estimates substantially, it remains difficult to exclude reverse causality given the long preclinical phase of dementia [32]. In addition, because dementia typically occurs at older ages, survival bias and competing risks may play a role, potentially weakening relations [33,34]. Lastly, it is possible that systolic blood pressure and LDL-cholesterol play a smaller role in risk of dementia in CVD patients compared to those without established CVD, possibly due to treatment effects or differences in underlying pathophysiology. Other individual CVD risk factors examined in the present study (i.e., excessive alcohol use, smoking, physical inactivity, obesity, inflammation, and albuminuria) showed hazard ratios between 1.05 and 1.28, which is consistent in direction of effect to that found in populations without established CVD [[13], [14], [15], [16], [17]].

Our findings have two main implications. First, they indicate that even after the onset of CVD, modifiable CVD risk factors remain relevant for long-term brain health. This underscores the importance of stringent cardiovascular risk management in individuals with established CVD, not only to prevent progression of CVD, but also as a potential strategy to reduce the risk of dementia. Although cardiovascular risk management is an important part of routine practice in individuals with CVD, adherence to secondary prevention guidelines tends to drop following the first few months after the initial CVD event [35,36]. As dementia is often the result of a gradual build-up of neuropathology, sustained, long-term multidomain intervention may be needed to reduce dementia risk. In line with this, the U.S. POINTER trial showed that a structured, higher-intensity multidomain lifestyle intervention improved global cognition in older adults at risk of cognitive decline compared with a self-guided intervention, supporting the potential of such an approach to prevent cognitive decline and possibly dementia [37]. Second, as those with a high CVD risk factor burden and higher number of affected vascular beds are at a higher risk of dementia, earlier or more proactive cognitive screening may be warranted in this population [38]. This may support personalized management strategies by enabling more targeted monitoring and interventions of these high-risk individuals.

Previous studies found that early onset (i.e., in midlife) compared to later onset of CVD and various CVD risk factors (e.g., type 2 diabetes, elevated systolic blood pressure, smoking and elevated LDL-cholesterol) are related to a higher risk of dementia [25,[39], [40], [41], [42], [43]]. We found no significant effect modification by age at onset in the relation between CVD risk factor burden and vascular bed involvement and dementia risk in our study population. This might be due to insufficient statistical power, as the majority of our study population (70%) were aged 40–65 at CVD onset, and this issue requires further study.

### 4.1 Strengths and limitations

Strengths of this study include a large sample size of patients recruited close to the time of their incident CVD event, with long-term follow-up and minimal attrition. Several limitations need to be considered as well. First, dementia ascertainment was undertaken through self-report questionnaires and medical record reviews, and we did not have cognitive data on the participants. This may have led to underreporting of dementia, particularly in individuals with incomplete follow-up. If non-differential, this would likely have led to an underestimation of true relations [44]. In addition, although follow-up was long (median 10.1 years [IQR 5.0–15.7], maximum 27 years), the mean age at baseline was relatively young (61 ± 10 years), which may have limited statistical power to assess incident dementia in the oldest age groups, where dementia incidence is highest. This may also have led to an underestimation of true relations. Second, data of blood pressure, LDL-cholesterol and medication use were only available at baseline and may have changed during follow-up. Third, no reliable data were available on dementia subtypes, and effects of risk factor prevalence and control may differ by the extent of co-pathology (e.g., Alzheimer’s disease in addition to vascular brain injury). Fourth, we cannot rule out residual confounding by premorbid risk factor exposure prior to baseline, or by reasons for which risk factor control at baseline was less stringent in some persons than in others. Fifth, our study population consisted of predominantly white, male, high-risk individuals with established CVD from a tertiary centre in the Netherlands with relatively good baseline risk factor control. The findings of this study may therefore be less generalisable to other CVD populations.

**Figure:**
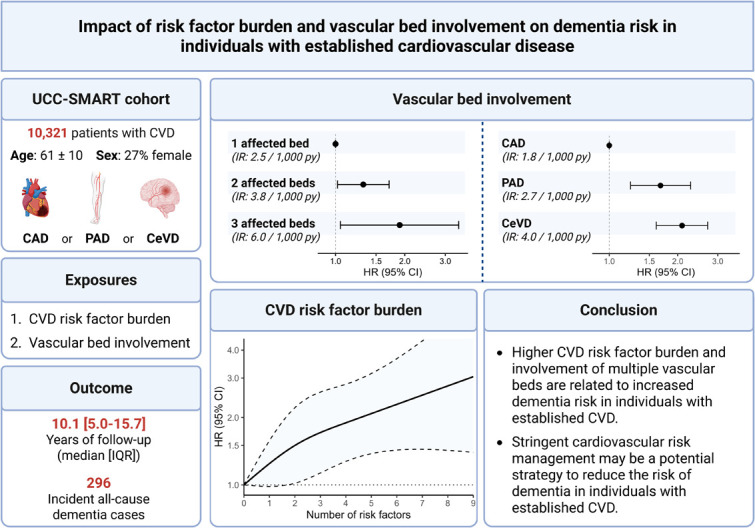
Central illustration.

Abbreviations: CAD - coronary artery disease, CeVD - cerebrovascular disease, CI - confidence interval, CVD - cardiovascular disease, HR - hazard ratio, IQR - interquartile range, IR - incidence rate, PAD - peripheral artery disease, PY - person-years, UCC-SMART - Utrecht Cardiovascular Cohort Second - Manifestations of Arterial Disease.

## 5 Conclusion

Higher CVD risk factor burden and involvement of multiple vascular beds are related to an increased risk of dementia in individuals with established CVD. These findings underscore the importance of stringent cardiovascular risk management in individuals with established CVD, not only to reduce the residual risk for recurrent CVD events, but also as a potential strategy to reduce the risk of dementia.

## Funding

Jan de Leijer and Thomas van Sloten are supported by grants from the European Foundation for the Study of Diabetes (EFSD) and the Dutch Diabetes Association.

## CRediT authorship contribution statement

**Jan F. de Leijer:** Writing – review & editing, Writing – original draft, Methodology, Data curation, Conceptualization. **Geert Jan Biessels:** Writing – review & editing, Supervision, Conceptualization. **Frank L.J. Visseren:** Writing – review & editing, Supervision, Conceptualization. **Tessa A.C.M. Vissers:** Writing – review & editing, Data curation. **Robin W.M. Vernooij:** Writing – review & editing, Supervision. **Frank J. Wolters:** Writing – review & editing, Methodology. **Sanaz Sedaghat:** Writing – review & editing. **Pamela L. Lutsey:** Writing – review & editing. **Mike J.L. Peters:** Writing – review & editing. **Archana Singh-Manoux:** Writing – review & editing, Methodology. **Thomas T. van Sloten:** Writing – review & editing, Writing – original draft, Supervision, Methodology, Conceptualization.

## Declaration of competing interest

The authors declare that they have no known competing financial interests or personal relationships that could have appeared to influence the work reported in this paper.

## Appendix Supplementary materials

Image, application 1
