# Supplementary material for: Impact of risk factor burden and vascular bed involvement on dementia risk in individuals with established cardiovascular disease
Source: Am J Prev Cardiol. 2026 Apr 17;29:101639. doi: 10.1016/j.ajpc.2026.101639 (PMC13329506; doi:10.1016/j.ajpc.2026.101639)
Supplement: Supplementary file 1 [file mmc1.docx]

# Supplemental materials

## Supplemental Table 1 - Baseline CVD subcategories

|  | Overall (n = 10,321) |
| --- | --- |
| **Cerebrovascular disease** | 3,378 (33) |
| Cerebral infarction | 1,999 (19) |
| Carotid artery stenosis >70% | 1,037 (10) |
| Carotid artery surgery or angioplasty | 395 (4) |
| **Coronary artery disease** | 6,480 (63) |
| Myocardial infarction | 3,479 (34) |
| Coronary artery bypass grafting or percutaneous transluminal coronary angioplasty | 4,931 (48) |
| **Peripheral artery disease** | 2,681 (26) |
| Ankle-brachial index ≤0.9 | 2,007 (19) |
| Lower-extremity vascular surgery or angioplasty | 584 (6) |
| Amputation | 108 (1) |

Values are count (%). Participants could be classified into more than one CVD category and subcategory at baseline; therefore, numbers within each CVD group do not sum to the overall total.

## Supplemental Table 2 - Baseline characteristics according to number of affected vascular beds

|  | Overall (n = 10,321) | 1 affected vascular bed (n = 8,368) | 2 affected vascular beds (n = 1,688) | 3 affected vascular beds (n = 265) |
| --- | --- | --- | --- | --- |
| Male sex | 7,531 (73) | 6,076 (73) | 1,237 (73) | 218 (82) |
| Age (years) | 61 ± 10 | 60 ± 10 | 65 ± 9 | 66 ± 9 |
| Current alcohol use | 6,028 (58) | 5,058 (60) | 861 (51) | 109 (41) |
| Current smoking | 2,900 (28) | 2,222 (27) | 573 (34) | 105 (40) |
| Packyears | 13 (1 - 31) | 12 (0 - 28) | 21 (8 - 37) | 28 (11 - 42) |
| Physical activity (METh/week) | 45 ± 41 | 46 ± 40 | 39 ± 41 | 35 ± 41 |
| Education level |  |  |  |  |
| Low | 3,425 (33) | 2,679 (32) | 646 (38) | 100 (38) |
| Middle | 3,515 (34) | 2,802 (34) | 612 (36) | 101 (38) |
| High | 3,381 (33) | 2,887 (35) | 430 (26) | 64 (24) |
| Affected vascular beds |  |  |  |  |
| Coronary artery disease | 6,480 (63) | 4,987 (60) | 1,228 (73) | 265 (100) |
| Cerebrovascular disease | 3,378 (33) | 2,101 (25) | 1,012 (60) | 265 (100) |
| Peripheral artery disease | 2,681 (26) | 1,280 (15) | 1,136 (67) | 265 (100) |
| Type 2 diabetes | 1,813 (18) | 1,304 (16) | 427 (25) | 82 (31) |
| Medication use |  |  |  |  |
| Blood pressure-lowering | 7,922 (77) | 6,307 (75) | 1,395 (83) | 220 (83) |
| Lipid-lowering | 7,438 (72) | 5,966 (71) | 1,264 (75) | 208 (79) |
| Antithrombotic | 8,700 (84) | 6,926 (83) | 1,535 (91) | 239 (90) |
| Anthropometric measurements |  |  |  |  |
| Body mass index (kg/m^2^) | 27 ± 4 | 27 ± 4 | 27 ± 4 | 27 ± 4 |
| Waist-to-hip ratio (F) | 0.85 ± 0.08 | 0.85 ± 0.08 | 0.87 ± 0.07 | 0.90 ± 0.08 |
| Waist-to-hip ratio (M) | 0.95 ± 0.07 | 0.94 ± 0.07 | 0.96 ± 0.07 | 0.96 ± 0.06 |
| Systolic blood pressure (mmHg) | 138 ± 21 | 137 ± 20 | 143 ± 22 | 147 ± 24 |
| Diastolic blood pressure (mmHg) | 81 ± 11 | 81 ± 11 | 80 ± 12 | 78 ± 12 |
| APOE genotype |  |  |  |  |
| One ε4 allele | 3,540 (34) | 2,914 (35) | 549 (33) | 77 (29) |
| Two ε4 alleles | 219 (2) | 166 (2) | 45 (3) | 8 (3) |
| Laboratory measurements |  |  |  |  |
| eGFR (ml/min/1.73m^2^) | 82 ± 18 | 83 ± 18 | 77 ± 20 | 71 ± 21 |
| UACR (mg/mmol) | 0.8 (0.5 - 1.7) | 0.8 (0.4 - 1.6) | 1.1 (0.6 - 2.9) | 1.5 (0.7 - 4.1) |
| HbA1c (%) | 5.7 (5.4 - 6.0) | 5.6 (5.4 - 6.0) | 5.8 (5.5 - 6.2) | 5.8 (5.5 - 6.4) |
| Total cholesterol (mmol/L) | 4.7 ± 1.2 | 4.7 ± 1.2 | 4.7 ± 1.2 | 4.8 ± 1.1 |
| HDL-cholesterol (mmol/L) | 1.2 ± 0.4 | 1.2 ± 0.4 | 1.2 ± 0.4 | 1.1 ± 0.3 |
| LDL-cholesterol (mmol/L) | 2.7 ± 1.1 | 2.7 ± 1.0 | 2.7 ± 1.1 | 2.8 ± 1.0 |
| C-reactive protein (mg/L) | 2.0 (1.0 - 4.3) | 1.9 (0.9 - 4.0) | 2.7 (1.2 - 5.2) | 3.0 (1.6 - 5.8) |

Values are median (IQR), mean ± SD or count (%).

Abbreviations: CVD - cardiovascular disease, eGFR - estimated glomerular filtration rate, HbA1c - glycated haemoglobin, HDL - high-density lipoprotein, LDL - low-density lipoprotein, M - male, METh/week - metabolic equivalent of task hours per week, F - female, UACR - urine albumin/creatinine ratio.

## Supplemental Table 3 - Relation between individual CVD risk factors and incident dementia

|  | **All-cause dementia** (296 events / 10,321 individuals / 107,977 person-years) | | | |
| --- | --- | --- | --- | --- |
|  | Events /  individuals | Model 1 HR (95% CI) | Model 2 HR (95% CI) | Model 3 HR (95% CI) |
| Type 2 diabetes | 72 / 1,813 | 1.50 (1.15 - 1.95) | 1.54 (1.17 - 2.01) | 1.46 (1.10 - 1.92) |
| Current smoking | 65 / 2,900 | 1.37 (1.03 - 1.82) | 1.28 (0.96 - 1.72) | 1.25 (0.93 - 1.68) |
| Alcohol use ≥10 units per week | 55 / 1,911 | 1.13 (0.84 - 1.51) | 1.10 (0.82 - 1.50) | 1.13 (0.83 - 1.53) |
| Highest quartile systolic blood pressure (≥150 mmHg) | 106 / 2,634 | 0.98 (0.77 - 1.25) | 0.96 (0.76 - 1.23) | 0.92 (0.72 - 1.18) |
| Highest quartile LDL-cholesterol (≥3.3 mmol/L) | 86 / 2,602 | 0.97 (0.75 - 1.24) | 0.86 (0.64 - 1.14) | 0.88 (0.65 - 1.17) |
| Lowest quartile physical activity (≤16 METh/week) | 91 / 2,590 | 1.32 (1.03 - 1.69) | 1.33 (1.02 - 1.71) | 1.28 (0.98 - 1.67) |
| Highest quartile waist-to-hip ratio (F: ≥0.9, M: ≥1.0) | 86 / 2,782 | 1.20 (0.91 - 1.59) | 1.23 (0.93 - 1.62) | 1.14 (0.85 - 1.51) |
| Highest quartile C-reactive protein (≥4.3 mg/L) | 72 / 2,594 | 1.12 (0.86 - 1.47) | 1.15 (0.87 - 1.52) | 1.05 (0.80 - 1.40) |
| Highest quartile UACR (≥1.7 mg/mmol) | 95 / 2,611 | 1.29 (1.01 - 1.66) | 1.25 (0.97 - 1.63) | 1.19 (0.92 - 1.56) |

Hazard ratios with 95% confidence intervals as derived from Cox proportional hazards models adjusted for age (Model 1), with additional adjustment for sex, education level, APOE genotype, years since first CVD diagnosis, atrial fibrillation, and blood pressure-lowering, lipid-lowering and antithrombotic medication (Model 2), and with mutual adjustment (Model 3). The reference group for each exposure consisted of all participants without the listed characteristic (e.g., without type 2 diabetes or systolic blood pressure <150 mmHg).

Abbreviations: CVD - cardiovascular disease, F - female, LDL - low-density lipoprotein, M - male, METh/week - metabolic equivalent of task hours per week, UACR - urine albumin/creatinine ratio.

## Supplemental Table 4 - Relation between CVD risk factor burden using standard clinical cut-offs and incident dementia

|  | **All-cause dementia** (296 events / 10,321 individuals / 107,977 person-years) | | |
| --- | --- | --- | --- |
|  | Events /  individuals | Model 1 HR (95% CI) | Model 2 HR (95% CI) |
| **CVD risk factor burden** |  |  |  |
| 0-2 risk factors | 44 / 2,114 | ref | ref |
| 3 risk factors | 75 / 2,723 | 1.19 (0.81 - 1.75) | 1.19 (0.80 - 1.76) |
| 4 risk factors | 76 / 2,723 | 1.30 (0.88 - 1.93) | 1.32 (0.89 - 1.96) |
| 5-9 risk factors | 101 / 2,761 | 1.84 (1.27 - 2.65) | 1.83 (1.26 - 2.65) |
|  |  |  |  |
| **Individual risk factors** |  |  |  |
| Type 2 diabetes | 72 / 1,813 | 1.50 (1.15 - 1.95) | 1.54 (1.17 - 2.01) |
| Current smoking | 65 / 2,900 | 1.37 (1.03 - 1.82) | 1.28 (0.96 - 1.72) |
| Alcohol use ≥10 units per week | 55 / 1,911 | 1.13 (0.84 - 1.51) | 1.10 (0.82 - 1.50) |
| Systolic blood pressure ≥140 mmHg | 157 / 4,368 | 0.90 (0.72 - 1.14) | 0.88 (0.70 - 1.11) |
| LDL-cholesterol ≥1.8 mmol/L | 252 / 8,384 | 0.98 (0.71 - 1.35) | 0.89 (0.64 - 1.24) |
| Elevated waist-to-hip ratio (F: ≥0.85, M: ≥0.9) | 218 / 7,422 | 1.07 (0.81 - 1.40) | 1.12 (0.84 - 1.48) |
| Physical activity ≤10 METh/week | 69 / 1,669 | 1.48 (1.13 - 1.94) | 1.51 (1.14 - 2.01) |
| C-reactive protein ≥3.0 mg/L | 102 / 3,840 | 1.02 (0.80 - 1.31) | 1.05 (0.82 - 1.35) |
| UACR ≥3.0 mg/mmol | 55 / 1,534 | 1.24 (0.92 - 1.68) | 1.19 (0.87 - 1.63) |

Hazard ratios with 95% confidence intervals as derived from Cox proportional hazards models adjusted for age (Model 1) and with additional adjustment for sex, education level, APOE genotype, years since first CVD diagnosis, atrial fibrillation, and blood pressure-lowering, lipid-lowering and antithrombotic medication (Model 2). The reference group for each exposure consisted of all participants without the listed characteristic (e.g., without type 2 diabetes or systolic blood pressure <140 mmHg).

Abbreviations: CVD - cardiovascular disease, F - female, LDL - low-density lipoprotein, M - male, METh/week - metabolic equivalent of task hours per week, UACR - urine albumin/creatinine ratio.

## Supplemental Table 5 - Relation between CVD risk factor burden and incident dementia adjusted for interim stroke

|  | **All-cause dementia** (296 events / 10,321 participants / 107,977 person-years) | | |
| --- | --- | --- | --- |
|  | Events /  individuals | Model 2 HR (95% CI) | Model 2 + adjustment for interim stroke HR (95% CI) |
| **CVD risk factor burden** |  |  |  |
| 0-1 risk factors | 52 / 2,669 | ref | ref |
| 2 risk factors | 77 / 2,764 | 1.35 (0.95 - 1.92) | 1.35 (0.95 - 1.92) |
| 3 risk factors | 78 / 2,351 | 1.58 (1.10 - 2.26) | 1.54 (1.08 - 2.21) |
| 4-9 risk factors | 89 / 2,537 | 1.87 (1.31 - 2.67) | 1.84 (1.29 - 2.63) |
|  |  |  |  |
| **Interim stroke** |  |  |  |
| No | 258 / 9,777 | - | ref |
| Yes | 38 / 592 | - | 2.43 (1.64 - 3.60) |

Hazard ratios (95% CI) for the relation between CVD risk factor burden and incident dementia with and without adjustment for interim stroke. Interim stroke was modeled as a time-varying covariate. This way, participants who had an interim stroke were analyzed in the group without an interim stroke until they had the interim stroke, after which they were moved to the group with an interim stroke for the remainder of follow-up. Models were adjusted for age, sex, education level, APOE genotype, years since first CVD diagnosis, atrial fibrillation, and blood pressure-lowering, lipid-lowering and antithrombotic medication (Model 2).

## Supplemental Table 6 - Relation between CVD risk factor burden, vascular bed involvement, and incident dementia with and without adjustment for APOE genotype

|  | Individuals with APOE genotype available | |
| --- | --- | --- |
|  | (n = 5,544, 225 events) | |
|  | Model 2 without adjustment for APOE genotype HR (95% CI) | Model 2 with adjustment for APOE genotype HR (95% CI) |
| **CVD risk factor burden** |  |  |
| 0-1 risk factors | ref | ref |
| 2 risk factors | 1.37 (0.91 – 2.08) | 1.40 (0.93 – 2.12) |
| 3 risk factors | 1.68 (1.11 – 2.53) | 1.71 (1.13 – 2.59) |
| 4-9 risk factors | 2.10 (1.40 – 3.16) | 2.14 (1.42 – 3.23) |
|  |  |  |
| **Number of affected vascular beds** |  |  |
| 1 affected vascular bed | ref | ref |
| 2 affected vascular beds | 1.27 (0.95 – 1.70) | 1.28 (0.96 – 1.71) |
| 3 affected vascular beds | 1.66 (0.81 – 3.40) | 1.77 (0.86 – 3.63) |
|  |  |  |
| **Individual affected vascular beds** |  |  |
| Coronary artery disease | ref | ref |
| Peripheral artery disease | 1.67 (1.15 – 2.43) | 1.67 (1.15 – 2.44) |
| Cerebrovascular disease | 1.88 (1.41 – 2.52) | 1.89 (1.41 – 2.53) |

Hazard ratios with 95% confidence intervals from Cox proportional hazards models derived in a subset of the full study population with APOE genotype available with and without adjustment for APOE genotype on top of adjustment for age, sex, education level, years since first CVD diagnosis, atrial fibrillation, and blood pressure-lowering, lipid-lowering and antithrombotic medication.

## Supplemental Table 7 - Relation between CVD risk factor burden, vascular bed involvement, and incident dementia according to median-split age at CVD onset groups

|  | Full study population | Age of CVD onset ≤57.5 | Age of CVD onset >57.5 | P for interaction with age of CVD onset |
| --- | --- | --- | --- | --- |
|  | (n = 10,321, 296 events) | (n = 5,171, 66 events) | (n = 5,150, 230 events) |  |
|  | Model 2 HR (95% CI) | Model 2 HR (95% CI) | Model 2 HR (95% CI) |  |
| **CVD risk factor burden** |  |  |  |  |
| 0-1 risk factors | ref | ref | ref | 0.08 |
| 2 risk factors | 1.35 (0.95 – 1.92) | 2.27 (1.02 – 5.07) | 1.11 (0.75 – 1.64) |  |
| 3 risk factors | 1.58 (1.10 – 2.26) | 2.59 (1.20 – 5.57) | 1.26 (0.85 – 1.87) |  |
| 4-9 risk factors | 1.87 (1.31 – 2.67) | 3.14 (1.44 – 6.82) | 1.74 (1.18 – 2.57) |  |
|  |  |  |  |  |
| **Number of affected vascular beds** |  |  |  |  |
| 1 affected vascular bed | ref | ref | ref | 0.78 |
| 2 affected vascular beds | 1.32 (1.02 – 1.71) | 1.25 (0.74 – 2.12) | 1.37 (1.03 – 1.83) |  |
| 3 affected vascular beds | 1.90 (1.05 – 3.43) | 2.10 (0.81 – 5.39) | 1.82 (0.85 – 3.90) |  |
|  |  |  |  |  |
| **Individual affected vascular beds** |  |  |  |  |
| Coronary artery disease | ref | ref | ref | 0.49 |
| Peripheral artery disease | 1.68 (1.24 – 2.28) | 1.19 (0.63 – 2.24) | 1.89 (1.33 – 2.69) |  |
| Cerebrovascular disease | 2.09 (1.61 – 2.71) | 2.29 (1.34 – 3.90) | 2.07 (1.53 – 2.81) |  |

Hazard ratios with 95% confidence intervals as derived from Cox proportional hazards models adjusted for age, sex, education level, APOE genotype, years since first CVD diagnosis, atrial fibrillation, and blood pressure-lowering, lipid-lowering and antithrombotic medication (Model 2).

## Supplemental Table 8 - Relation between CVD risk factor burden, vascular bed involvement, and incident dementia according to sex

|  | Full study population | Men | Women | P for interaction with sex |
| --- | --- | --- | --- | --- |
|  | (n = 10,321, 296 events) | (n = 7,531, 212 events) | (n = 2,790, 84 events) |  |
|  | Model 2 HR (95% CI) | Model 2 HR (95% CI) | Model 2 HR (95% CI) |  |
| **CVD risk factor burden** |  |  |  |  |
| 0-1 risk factors | ref | ref | ref | 0.89 |
| 2 risk factors | 1.35 (0.95 - 1.92) | 1.38 (0.92 - 2.09) | 1.26 (0.63 - 2.55) |  |
| 3 risk factors | 1.58 (1.10 - 2.26) | 1.66 (1.10 - 2.51) | 1.34 (0.67 - 2.68) |  |
| 4-9 risk factors | 1.87 (1.31 - 2.67) | 2.03 (1.35 - 3.07) | 1.66 (0.83 - 3.30) |  |
|  |  |  |  |  |
| **Number of affected vascular beds** |  |  |  |  |
| 1 affected vascular bed | ref | ref | ref | 0.64 |
| 2 affected vascular beds | 1.32 (1.02 - 1.71) | 1.38 (1.03 - 1.87) | 1.20 (0.73 - 1.96) |  |
| 3 affected vascular beds | 1.90 (1.05 - 3.43) | 2.11 (1.13 - 3.94) | 1.06 (0.14 - 8.05) |  |
|  |  |  |  |  |
| **Individual affected vascular beds** |  |  |  |  |
| Coronary artery disease | ref | ref | ref | 0.75 |
| Peripheral artery disease | 1.68 (1.24 - 2.28) | 1.60 (1.12 - 2.29) | 2.02 (1.10 - 3.71) |  |
| Cerebrovascular disease | 2.09 (1.61 - 2.71) | 2.39 (1.77 - 3.23) | 1.54 (0.94 - 2.51) |  |

Hazard ratios with 95% confidence intervals as derived from Cox proportional hazards models adjusted for age, sex, education level, APOE genotype, years since first CVD diagnosis, atrial fibrillation, and blood pressure-lowering, lipid-lowering and antithrombotic medication (Model 2).

## Supplemental Table 9 - Relation between CVD risk factor burden and incident dementia according to baseline affected vascular bed

|  | Coronary artery disease | Peripheral artery disease | Cerebrovascular disease | P for interaction with baseline affected vascular bed |
| --- | --- | --- | --- | --- |
|  | (n = 6,480, 150 events) | (n = 2,681, 87 events) | (n = 3,378, 140 events) |  |
|  | Model 2 HR (95% CI) | Model 2 HR (95% CI) | Model 2 HR (95% CI) |  |
| **CVD risk factor burden** |  |  |  |  |
| 0-1 risk factors | ref | ref | ref | ≥0.20* |
| 2 risk factors | 1.46 (0.91 – 2.33) | 1.48 (0.65 – 3.36) | 1.30 (0.78 – 2.19) |  |
| 3 risk factors | 1.81 (1.12 – 2.92) | 1.67 (0.71 – 3.88) | 1.31 (0.78 – 2.21) |  |
| 4-9 risk factors | 2.10 (1.29 – 3.43) | 1.67 (0.70 – 3.98) | 1.68 (1.00 – 2.85) |  |

Hazard ratios with 95% confidence intervals as derived from Cox proportional hazards models adjusted for age, sex, education level, APOE genotype, years since first CVD diagnosis, atrial fibrillation, and blood pressure-lowering, lipid-lowering and antithrombotic medication (Model 2).

*P values for interaction were obtained by comparing, using a likelihood ratio test, a model including the respective vascular bed as a covariate with a model additionally including a multiplicative interaction term between CVD risk factor burden and the respective vascular bed. Interaction P values were 0.64 for cerebrovascular disease, 0.20 for peripheral artery disease, and 0.47 for coronary artery disease.

## Supplemental Table 10 - Relation between CVD risk factor burden, vascular bed involvement, and incident dementia according to APOE genotype

|  | Full study population | No ε4 alleles | At least one ε4 allele | P for interaction with APOE genotype |
| --- | --- | --- | --- | --- |
|  | (n = 10,321, 296 events) | (n = 6,562, 163 events) | (n = 3,759, 133 events) |  |
|  | Model 2 HR (95% CI) | Model 2 HR (95% CI) | Model 2 HR (95% CI) |  |
| **CVD risk factor burden** |  |  |  |  |
| 0-1 risk factors | ref | ref | ref | 0.43 |
| 2 risk factors | 1.35 (0.95 – 1.92) | 1.75 (1.06 – 2.90) | 1.08 (0.66 – 1.79) |  |
| 3 risk factors | 1.58 (1.10 – 2.26) | 2.02 (1.21 – 3.56) | 1.21 (0.73 – 2.01) |  |
| 4-9 risk factors | 1.87 (1.31 – 2.67) | 2.60 (1.57 – 4.30) | 1.45 (0.87 – 2.41) |  |
|  |  |  |  |  |
| **Number of affected vascular beds** |  |  |  |  |
| 1 affected vascular bed | ref | ref | ref | 0.63 |
| 2 affected vascular beds | 1.32 (1.02 – 1.71) | 1.22 (0.87 – 1.73) | 1.44 (0.96 – 2.16) |  |
| 3 affected vascular beds | 1.90 (1.05 – 3.43) | 2.04 (0.94 – 4.43) | 1.44 (0.31 – 6.74) |  |
|  |  |  |  |  |
| **Individual affected vascular beds** |  |  |  |  |
| Coronary artery disease | ref | ref | ref | 0.22 |
| Peripheral artery disease | 1.68 (1.24 – 2.28) | 1.77 (1.06 – 2.96) | 1.56 (0.91 – 2.66) |  |
| Cerebrovascular disease | 2.09 (1.61 – 2.71) | 2.29 (1.61 – 3.26) | 1.84 (1.24 – 2.73) |  |

Hazard ratios with 95% confidence intervals as derived from Cox proportional hazards models adjusted for age, sex, education level, APOE genotype, years since first CVD diagnosis, atrial fibrillation, and blood pressure-lowering, lipid-lowering and antithrombotic medication (Model 2).

## Supplemental Table 11 - Reverse causality assessment

|  | Full study population | Exclude events <1 year | Exclude events <5 year | Exclude events <10 year |
| --- | --- | --- | --- | --- |
| Number of events / individuals | 296 / 10,321 | 290 / 10,315 | 238 / 10,263 | 150 / 10,175 |
| **CVD risk factor burden** |  |  |  |  |
| 0-1 risk factors | ref | ref | ref | ref |
| 2 risk factors, HR (95% CI) | 1.35 (0.95 - 1.92) | 1.40 (0.99 - 1.98) | 1.39 (0.94 - 2.06) | 1.60 (0.95 - 2.70) |
| 3 risk factors, HR (95% CI) | 1.58 (1.10 - 2.26) | 1.55 (1.09 - 2.20) | 1.45 (0.98 - 2.16) | 1.63 (0.98 - 2.72) |
| 4-9 risk factors, HR (95% CI) | 1.87 (1.31 - 2.67) | 1.85 (1.30 - 2.65) | 1.89 (1.28 - 2.80) | 2.05 (1.22 - 3.45) |
|  |  |  |  |  |
| **Number of affected vascular beds** |  |  |  |  |
| 1 affected vascular bed, HR (95% CI) | ref | ref | ref | ref |
| 2 affected vascular beds, HR (95% CI) | 1.32 (1.02 - 1.71) | 1.30 (1.00 - 1.77) | 1.34 (0.94 - 1.92) | 1.06 (0.76 - 1.49) |
| 3 affected vascular beds, HR (95% CI) | 1.90 (1.05 - 3.43) | 1.95 (1.13 - 3.37) | 1.83 (0.89 - 3.77) | 1.43 (0.70 - 2.92) |
|  |  |  |  |  |
| **Individual affected vascular beds** |  |  |  |  |
| Coronary artery disease | ref | ref | ref | ref |
| Peripheral artery disease | 1.68 (1.24 - 2.28) | 1.72 (1.27 - 2.33) | 1.63 (1.16 - 2.30) | 1.20 (0.77 - 1.88) |
| Cerebrovascular disease | 2.09 (1.61 - 2.71) | 2.09 (1.60 - 2.73) | 2.15 (1.60 - 2.90) | 1.64 (1.13 - 2.37) |

Hazard ratios for incident dementia after excluding participants who were diagnosed with dementia within the first 1, 5 and 10 years of inclusion. Models were adjusted for age, sex, education level, APOE genotype, years since first CVD diagnosis, atrial fibrillation, and blood pressure-lowering, lipid-lowering and antithrombotic medication (Model 2).

## Supplemental Table 12 - Relation between CVD risk factor burden and incident dementia adjusted for medication use at baseline

|  | **All-cause dementia** | | |
| --- | --- | --- | --- |
|  | (296 events / 10,321 individuals / 107,977 person-years) | | |
|  | Events / | Model 1 | Model 2 |
|  | individuals | HR (95% CI) | HR (95% CI) |
| **CVD risk factor burden** |  |  |  |
| 0-1 risk factors | 57 / 2,661 | ref | ref |
| 2 risk factors | 75 / 2,809 | 1.29 (0.92 - 1.81) | 1.30 (0.92 - 1.82) |
| 3 risk factors | 72 / 2,304 | 1.50 (1.11 - 2.05) | 1.51 (1.11 - 2.06) |
| 4-9 risk factors | 92 / 2,547 | 1.88 (1.35 - 2.62) | 1.89 (1.34 - 2.66) |
|  |  |  |  |
| **Individual risk factors** |  |  |  |
| Type 2 diabetes | 72 / 1,813 | 1.50 (1.15 - 1.95) | 1.54 (1.17 - 2.01) |
| Current smoking | 65 / 2,900 | 1.37 (1.03 - 1.82) | 1.28 (0.96 - 1.72) |
| Alcohol use ≥10 units per week | 55 / 1,911 | 1.13 (0.84 - 1.51) | 1.10 (0.82 - 1.50) |
| Highest quartile systolic blood pressure (≥158 mmHg)* | 107 / 2,489 | 1.04 (0.82 - 1.33) | 1.07 (0.84 - 1.38) |
| Highest quartile LDL-cholesterol (≥3.8 mmol/L)* | 74 / 2,514 | 0.97 (0.75 - 1.24) | 0.86 (0.64 - 1.14) |
| Lowest quartile physical activity (≤16 METh/week) | 91 / 2,590 | 1.32 (1.03 - 1.69) | 1.33 (1.02 - 1.71) |
| Highest quartile waist-to-hip ratio (F: ≥0.9, M: ≥1.0) | 86 / 2,782 | 1.20 (0.91 - 1.59) | 1.23 (0.93 - 1.62) |
| Highest quartile C-reactive protein (≥4.3 mg/L) | 72 / 2,594 | 1.12 (0.86 - 1.47) | 1.15 (0.87 - 1.52) |
| Highest quartile UACR (≥1.7 mg/mmol) | 95 / 2,611 | 1.29 (1.01 - 1.66) | 1.25 (0.97 - 1.63) |

Hazard ratios with 95% confidence intervals as derived from Cox proportional hazards models adjusted for age (Model 1) and with additional adjustment for sex, education level, APOE genotype, years since first CVD diagnosis, atrial fibrillation, and blood pressure-lowering, lipid-lowering and antithrombotic medication (Model 2). The reference group for each exposure consisted of all participants without the listed characteristic (e.g., without type 2 diabetes or systolic blood pressure <158 mmHg).

*Systolic blood pressure and LDL-cholesterol were adjusted for the expected effects of blood pressure-lowering therapies (5.7-31.7 mmHg) and lipid-lowering therapies (24-49%) at baseline.

Abbreviations: CVD - cardiovascular disease, F - female, LDL - low-density lipoprotein, M - male, METh/week - metabolic equivalent of task hours per week, UACR - urine albumin/creatinine ratio.

## Supplemental Table 13 - Relation between CVD risk factor burden, vascular bed involvement, and incident dementia after exclusion of participants with prior stroke at baseline

|  | Full study population | Exclusion of individuals with stroke at baseline |
| --- | --- | --- |
|  | (n = 10,321, 296 events) | (n = 8,322, 223 events) |
|  | Model 2 HR (95% CI) | Model 2 HR (95% CI) |
| **CVD risk factor burden** |  |  |
| 0-1 risk factors | ref | ref |
| 2 risk factors | 1.35 (0.95 - 1.92) | 1.29 (0.85 - 1.96) |
| 3 risk factors | 1.58 (1.10 - 2.26) | 1.53 (1.02 - 2.32) |
| 4-9 risk factors | 1.87 (1.31 - 2.67) | 2.12 (1.41 - 3.18) |
|  |  |  |
| **Number of affected vascular beds** |  |  |
| 1 affected vascular bed | ref | ref |
| 2 affected vascular beds | 1.32 (1.02 - 1.71) | 1.30 (1.01 - 1.68) |
| 3 affected vascular beds | 1.90 (1.05 - 3.43) | 1.85 (1.03 - 3.33) |
|  |  |  |
| **Individual affected vascular beds** |  |  |
| Coronary artery disease | ref | ref |
| Peripheral artery disease | 1.68 (1.24 - 2.28) | 1.68 (1.24 - 2.28) |
| Cerebrovascular disease | 2.09 (1.61 - 2.71) | 1.66 (1.24 - 2.23) |

Hazard ratios with 95% confidence intervals as derived from Cox proportional hazards models adjusted for age, sex, education level, APOE genotype, years since first CVD diagnosis, atrial fibrillation, and blood pressure-lowering, lipid-lowering and antithrombotic medication (Model 2).

## Supplemental Table 14 - Relation between CVD risk factor burden, vascular bed involvement, and incident dementia with additional adjustment for year of inclusion

|  | **All-cause dementia** (296 events / 10,321 individuals / 107,977 person-years) | |
| --- | --- | --- |
|  | Model 2 without adjustment for inclusion year HR (95% CI) | Model 2 with adjustment for inclusion year HR (95% CI) |
| **CVD risk factor burden** |  |  |
| 0-1 risk factors | ref | ref |
| 2 risk factors | 1.35 (0.95 - 1.92) | 1.35 (0.95 - 1.92) |
| 3 risk factors | 1.58 (1.10 - 2.26) | 1.58 (1.10 - 2.27) |
| 4-9 risk factors | 1.87 (1.31 - 2.67) | 1.88 (1.32 - 2.68) |
|  |  |  |
| **Number of affected vascular beds** |  |  |
| 1 affected vascular bed | ref | ref |
| 2 affected vascular beds | 1.32 (1.02 - 1.71) | 1.34 (1.04 - 1.74) |
| 3 affected vascular beds | 1.90 (1.05 - 3.43) | 1.94 (1.07 - 3.51) |
|  |  |  |
| **Individual affected vascular beds** |  |  |
| Coronary artery disease | ref | ref |
| Peripheral artery disease | 1.68 (1.24 - 2.28) | 1.68 (1.19 - 2.37) |
| Cerebrovascular disease | 2.09 (1.61 - 2.71) | 2.22 (1.66 - 2.97) |

Hazard ratios with 95% confidence intervals as derived from Cox proportional hazards models with and without adjustment for year of inclusion on top of adjustment for age, sex, education level, APOE genotype, years since first CVD diagnosis, atrial fibrillation, and blood pressure-lowering, lipid-lowering and antithrombotic medication.

## Supplemental Table 15 - Relation between CVD risk factor burden, vascular bed involvement, and incident dementia with additional adjustment for the competing risk of all-cause mortality

|  | **All-cause dementia** (296 events / 10,321 individuals / 107,977 person-years) | |
| --- | --- | --- |
|  | Cause-specific Cox-models HR (95% CI) | Fine and Gray models SHR (95% CI) |
| **CVD risk factor burden** |  |  |
| 0-1 risk factors | ref | ref |
| 2 risk factors | 1.35 (0.95 - 1.92) | 1.37 (0.96 - 1.95) |
| 3 risk factors | 1.58 (1.10 - 2.26) | 1.51 (1.06 - 2.16) |
| 4-9 risk factors | 1.87 (1.31 - 2.67) | 1.63 (1.17 - 2.28) |
|  |  |  |
| **Number of affected vascular beds** |  |  |
| 1 affected vascular bed | ref | ref |
| 2 affected vascular beds | 1.32 (1.02 - 1.71) | 1.44 (1.04 - 1.99) |
| 3 affected vascular beds | 1.90 (1.05 - 3.43) | 1.90 (1.04 - 3.49) |
|  |  |  |
| **Individual affected vascular beds** |  |  |
| Coronary artery disease | ref | ref |
| Peripheral artery disease | 1.68 (1.24 - 2.28) | 1.42 (1.03 - 1.95) |
| Cerebrovascular disease | 2.09 (1.61 - 2.71) | 2.12 (1.58 - 2.85) |

Hazard ratios with 95% confidence intervals for all-cause dementia as derived from cause-specific Cox proportional hazards models and Fine and Gray subdistribution hazard models accounting for the competing risk of all-cause mortality, adjusted for age, sex, education level, APOE genotype, years since first CVD diagnosis, atrial fibrillation, and use of blood pressure-lowering, lipid-lowering, and antithrombotic medication (Model 2).

## Supplemental Figure 1


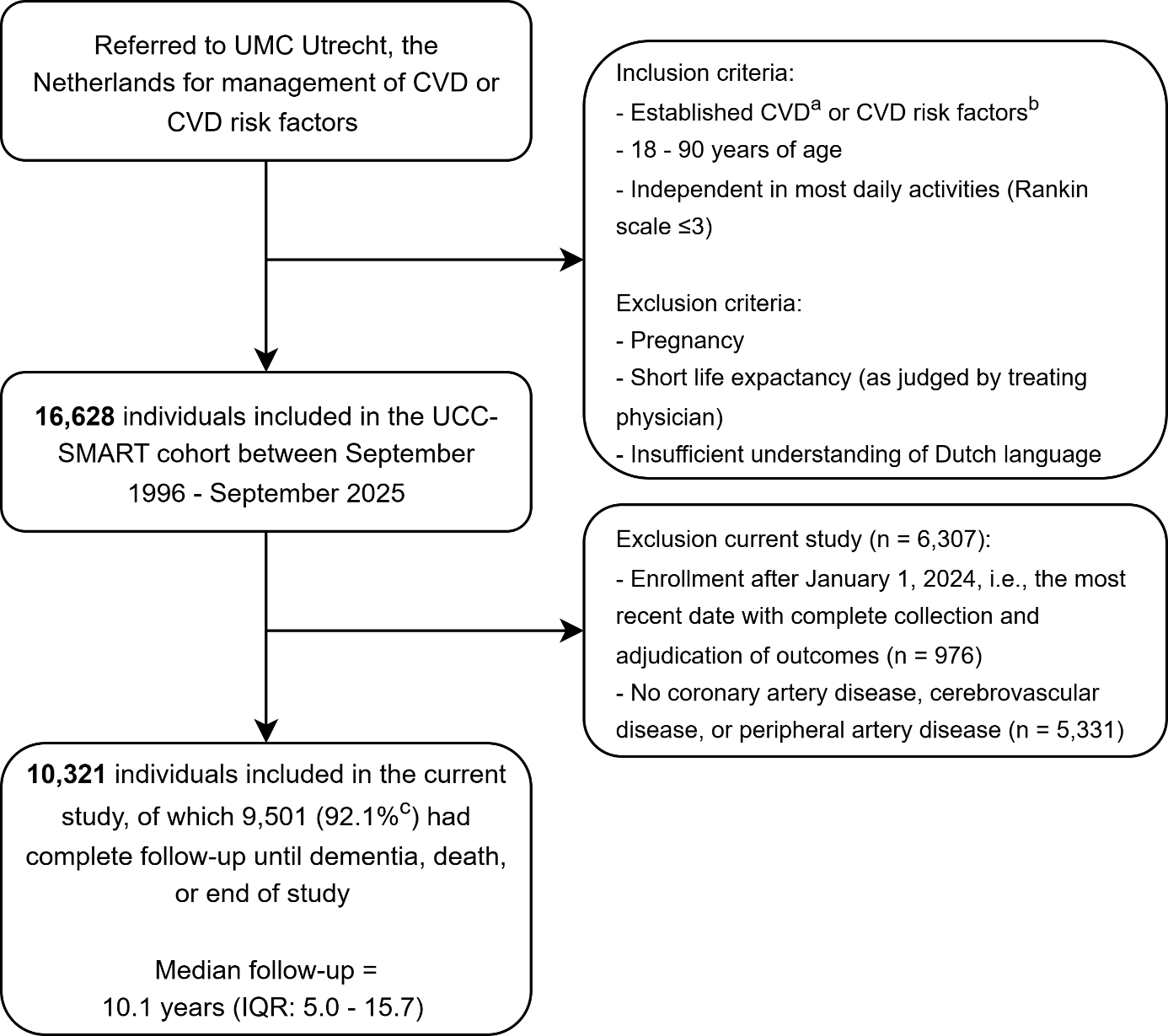


Flowchart of enrollment in the UCC-SMART cohort, and in the current study.

^a^Coronary artery disease (i.e., myocardial infarction, angina pectoris, ≥1 vessel disease on coronary angiography, or coronary revascularization in medical history), cerebrovascular disease (i.e., transient ischemic attack, cerebral infarction, carotid artery stenosis, ischemic retinal syndrome, carotid surgery or angioplasty in medical history), peripheral artery disease (i.e., Fontaine classification ≥II with ankle-brachial index ≤0.9, amputation, vascular surgery or angioplasty in medical history), abdominal aortic aneurysm with an anterior-posterior diameter ≥3 cm, or renal artery stenosis (≥ 50% narrowing of lumen caused by atherosclerosis in ≥ 1 renal artery).

^b^Hypertension, hyperlipidaemia, diabetes mellitus, renal insufficiency, chronic HIV infection, or a positive family medical history for cardiovascular disease (CVD).

^c^Median follow-up time for those without complete follow-up due to withdrawal of participation or by being unreachable for further questionnaires was 8.9 years (IQR 5.0 - 12.9).

Abbreviations: CVD - cardiovascular disease, UCC-SMART - Utrecht Cardiovascular Cohort Second - Manifestations of Arterial Disease, UMC Utrecht - University Medical Centre Utrecht

## Supplemental Figure 2


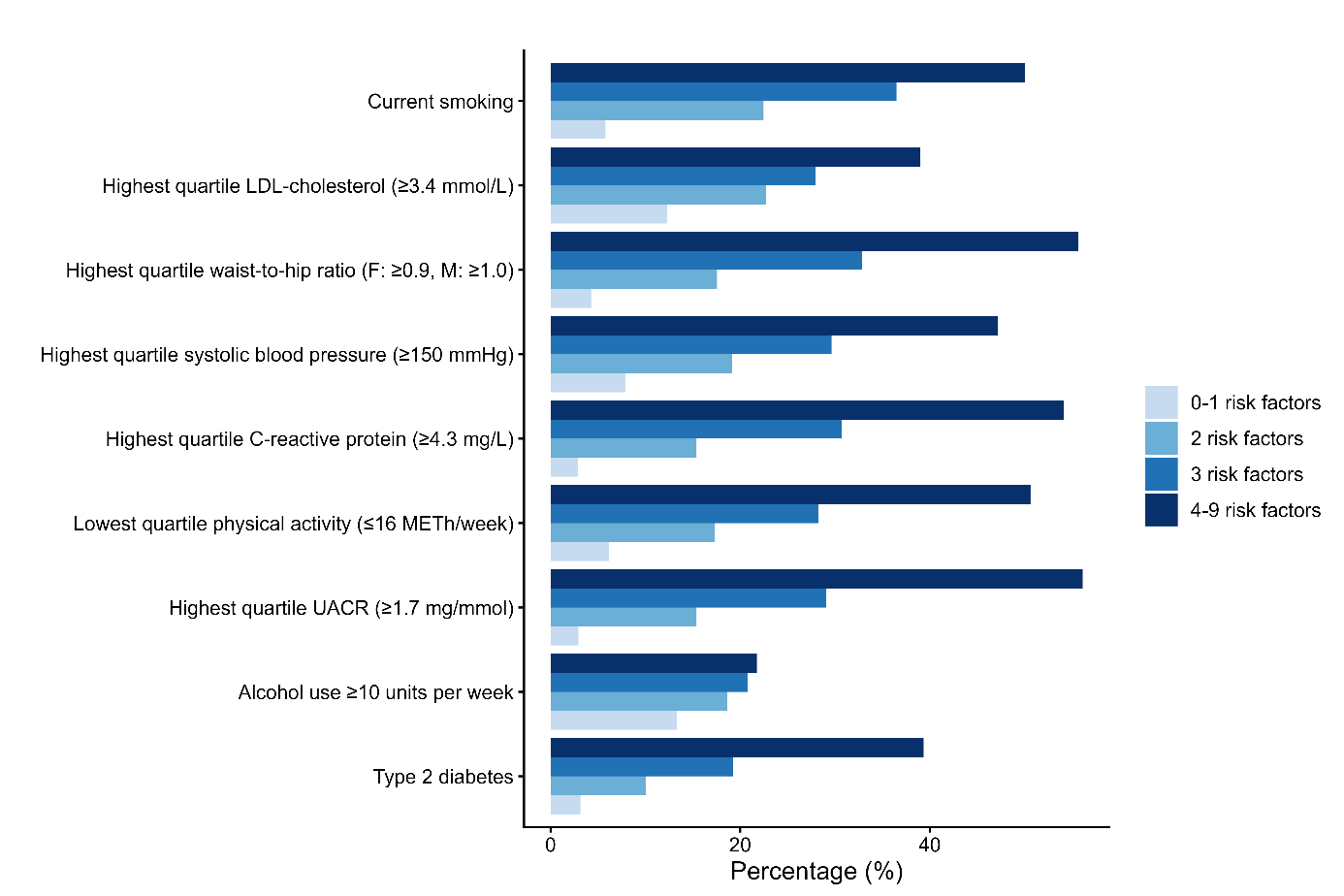


Proportions of dichotomized individual CVD risk factors according to CVD risk factor burden categories.

## Supplemental Figure 3


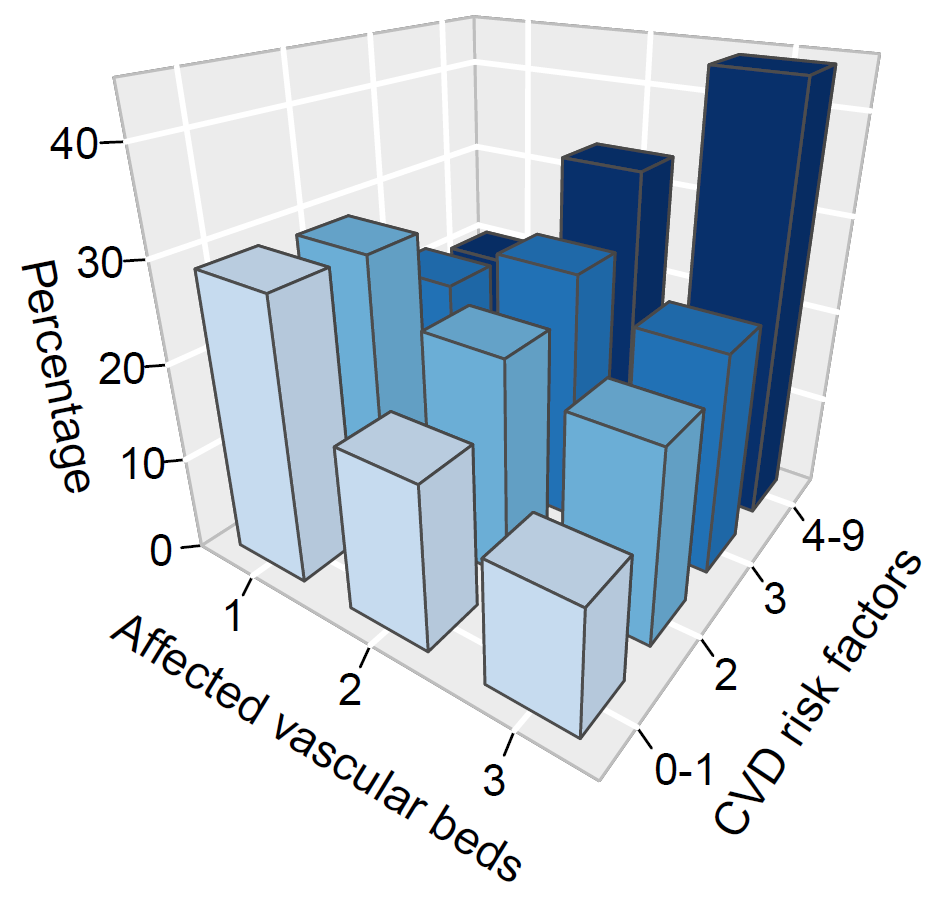


Proportions of CVD risk factor burden categories according to number affected vascular beds in participants with established CVD.

##
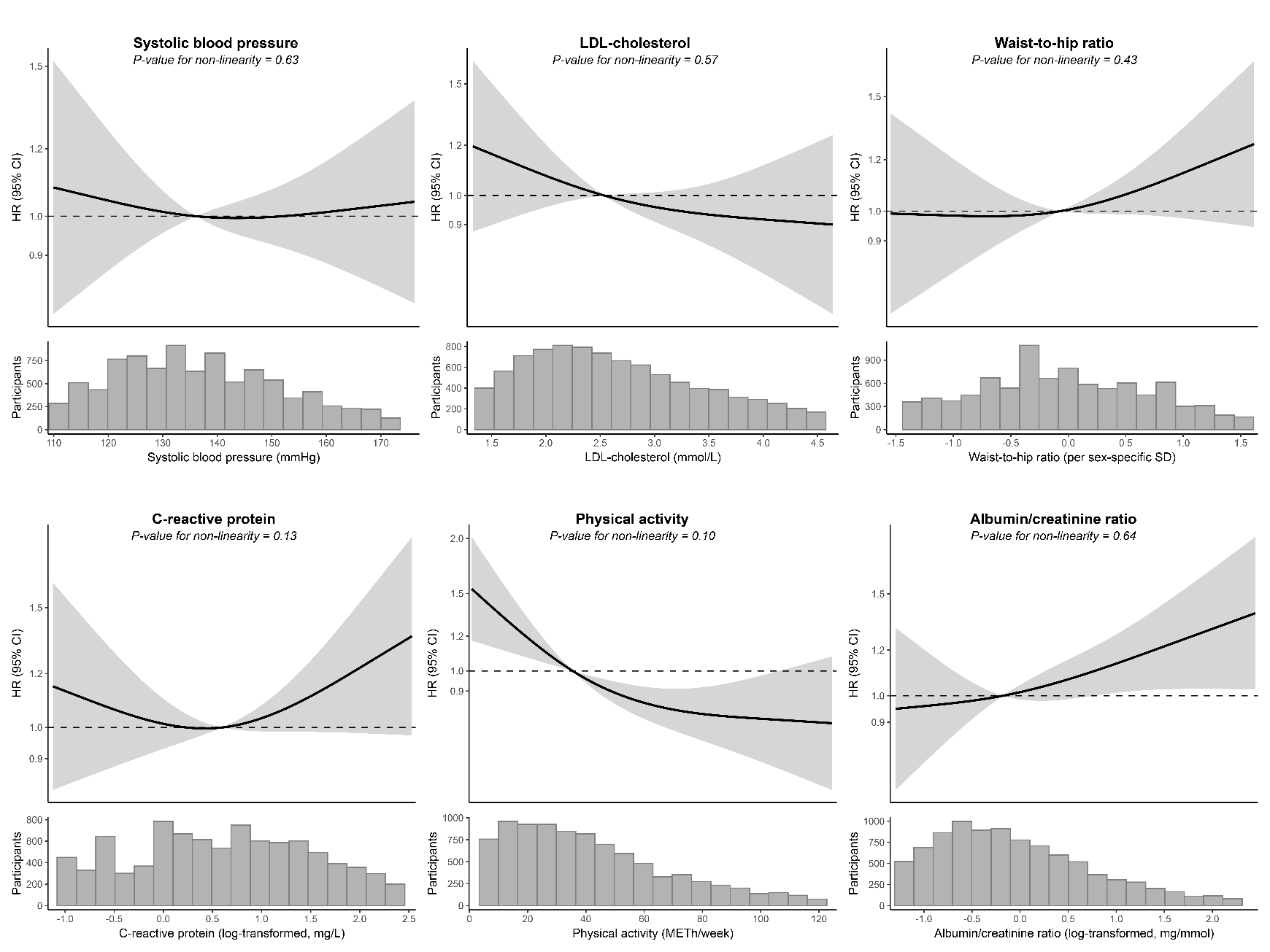
Supplemental Figure 4

Continuous relations between CVD risk factors and incident dementia. The figure presents the best-fitting restricted cubic splines for each risk factor, using three knots at the 10th, 50th, and 90th percentiles, with the reference value set at 1.0. Splines are adjusted for age, sex, education level, APOE genotype, years since first CVD diagnosis, atrial fibrillation and blood pressure-lowering, lipid-lowering and antithrombotic medication (Model 2).
